# Genome Sequence of the Intracellular Bacterium Wolbachia

**DOI:** 10.1371/journal.pbio.0020076

**Published:** 2004-03-16

Wolbachia have a thing against males. A member of one of the most diverse groups of bacteria, called Proteobacteria, this parasitic “endosymbiont” lives inside the reproductive cells of a wide variety of the nearly 1 million species of arthropods, including insects, spiders, and crustaceans. It has also been found in worms. Wolbachia's preferred habitat is the cytoplasm of its host's gametes. Since sperm have very little cytoplasm, Wolbachia seek out the company of females, securing its survival by hitching a ride to the next generation in the cytoplasm of the mother's eggs. Wolbachia's effects range from beneficial to pathological, depending on which species infects which invertebrate host, but since most species are not beneficial, Wolbachia infections often turn out badly if the host is male. On the other hand, if female, the host could very well live longer, produce more eggs, and have higher hatching rates than its noninfected cousins—thereby facilitating Wolbachia's transmission from mother to offspring.

Wolbachia have evolved an impressive repertoire of “reproductive parasitic” strategies to adapt its host's physiology to its own advantage. One strategy involves inducing “cytoplasmic incompatibility” between sperm and egg, which in effect uses infected males to keep uninfected females from producing viable offspring. Another causes infected females to reproduce asexually, creating a new generation of infected clones. Another turns developing male embryos into females. And, in a pinch, some Wolbachia simply kill developing males. The biochemical mechanisms that trigger different strategies in different hosts are unclear, however, in part because it's so far been impossible to cultivate sufficient quantities of these obligate endosymbionts (that is, intracellular species that cannot survive outside their host). But now that Scott O'Neill, Jonathan Eisen, and colleagues have sequenced the complete genome of one strain of Wolbachia pipientis, scientists investigating the biology and evolution of Wolbachia–host interactions have a valuable new research tool. The strain they sequenced, W. pipientis *w*Mel, lives inside the fruitfly Drosophila melanogaster, the favorite model organism of geneticists for nearly 100 years. This strain causes cytoplasmic incompatibility in its host.[Fig pbio-0020076-g001]

**Figure pbio-0020076-g001:**
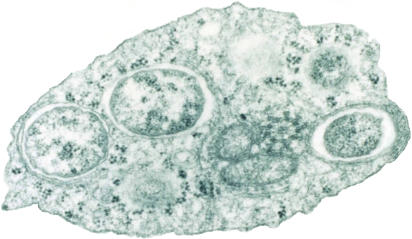
Transmission electron micrograph of Wolbachia within an insect cell (Image courtesy of Scott O'Neill)

The structure of the *w*Mel genome, the O'Neill and Eisen groups note, is strikingly different from any other obligate intracellular species. While its genome is compact, it nonetheless contains large amounts of repetitive DNA and “mobile” DNA elements. Mobile genetic elements, as the name implies, are DNA sequences that move around the genome and are often acquired from other species. Most of the repetitive and mobile elements in Wolbachia do not appear in other α-Proteobacteria species and were probably introduced some time after Wolbachia split off from its evolutionary ancestors. Wolbachia, unlike other obligate intracellular bacteria, seem quite amenable to incorporating foreign DNA, which the authors speculate was introduced by the bacteria-infecting virus called phage.

Analysis of the Wolbachia genome sheds light on the mechanisms that might help the parasite manipulate the host cell's physiology to its own advantage. One likely bacterial weapon for host exploitation is the abundance of predicted genes encoding ankyrin repeat domains, amino acid sequences characteristic of proteins important for protein–protein interactions in eukaryotes (organisms with nuclei, which bacteria lack). In bacteria, ankyrin repeats might regulate host cell-cycle pathways, which one wasp-infecting Wolbachia strain modifies to induce cytoplasmic incompatibility. Other molecular interactions between *w*Mel and its host, the researchers propose, might also rely on proteins with these ankyrin repeats.

The Wolbachia genome also provides insight into mitochondrial evolution. It is widely believed that these intracellular energy-metabolizing centers were once free-living bacteria belonging to the α-Proteobacteria group, though it's not clear which branch of the α-Proteobacteria tree they inhabit. Complete genome analysis of various α-Proteobacteria—including *w*Mel, the first non-Rickettsia species sequenced in the Rickettsiales group—provides no evidence that mitochondria are more related to Rickettsia species than to Wolbachia, as was previously thought. In fact, further analysis failed to consistently connect mitochondria to any particular species or group within the α-Proteobacteria.

While the information hidden in the Wolbachia genome seems to raise as many issues as it settles, biologists studying a wide range of problems—from the evolution and biology of Wolbachia and endosymbiont–host interactions to the origin of mitochondria—have a valuable new tool to explore their questions. The Wolbachia genome will also provide important molecular guidance for efforts to suppress insect pests and control filariasis, a human disease caused by worms. Since beneficial Wolbachia live in both insect and worm, applying antibiotics to target the Wolbachia will ultimately kill the insect pest and infecting worm, which both depend on the bacteria to survive.

